# TopoRoot+: computing whorl and soil line traits of field-excavated maize roots from CT imaging

**DOI:** 10.1186/s13007-024-01240-0

**Published:** 2024-08-27

**Authors:** Yiwen Ju, Alexander E. Liu, Kenan Oestreich, Tina Wang, Christopher N. Topp, Tao Ju

**Affiliations:** 1 Washington University in Saint Louis, St. Louis, USA; 2 Donald Danforth Plant Science Center, Creve Coeur, USA; 3 Marquette High School, Chesterfield, MO USA

**Keywords:** Root system architecture, Maize phenotyping, 3D imaging, Computer graphics

## Abstract

**Background:**

The use of 3D imaging techniques, such as X-ray CT, in root phenotyping has become more widespread in recent years. However, due to the complexity of the root structure, analyzing the resulting 3D volumes to obtain detailed architectural root traits remains a challenging computational problem. When it comes to image-based phenotyping of excavated maize root crowns, two types of root features that are notably missing from existing methods are the whorls and soil line. Whorls refer to the distinct areas located at the base of each stem node from which roots sprout in a circular pattern (Liu S, Barrow CS, Hanlon M, Lynch JP, Bucksch A. Dirt/3D: 3D root phenotyping for field-grown maize (*zea mays*). Plant Physiol. 2021;187(2):739–57. 10.1093/plphys/kiab311.). The soil line is where the root stem meets the ground. Knowledge of these features would give biologists deeper insights into the root system architecture (RSA) and the below- and above-ground root properties.

**Results:**

We developed TopoRoot+, a computational pipeline that produces architectural traits from 3D X-ray CT volumes of excavated maize root crowns. Building upon the TopoRoot software (Zeng D, Li M, Jiang N, Ju Y, Schreiber H, Chambers E, et al. Toporoot: A method for computing hierarchy and fine-grained traits of maize roots from 3D imaging. Plant Methods. 2021;17(1). 10.1186/s13007-021-00829-z.) for computing fine-grained root traits, TopoRoot + adds the capability to detect whorls, identify nodal roots at each whorl, and compute the soil line location. The new algorithms in TopoRoot + offer an additional set of fine-grained traits beyond those provided by TopoRoot. The addition includes internode distances, root traits at every hierarchy level associated with a whorl, and root traits specific to above or below the ground. TopoRoot + is validated on a diverse collection of field-grown maize root crowns consisting of nine genotypes and spanning across three years. TopoRoot + runs in minutes for a typical volume size of $\:40{0}^{3}$ on a desktop workstation. Our software and test dataset are freely distributed on Github.

**Conclusions:**

TopoRoot + advances the state-of-the-art in image-based phenotyping of excavated maize root crowns by offering more detailed architectural traits related to whorls and soil lines. The efficiency of TopoRoot + makes it well-suited for high-throughput image-based root phenotyping.

**Supplementary Information:**

The online version contains supplementary material available at 10.1186/s13007-024-01240-0.

## Introduction

Roots offer key services to both the plant and the environment. They provide anchorage for the plant, extract water and nutrients from the soil, and sequester carbon from the atmosphere. The root system architecture (RSA), which describes the hierarchical organization of roots, has profound implications on how well roots perform these services [1–3]. Quantifying the RSA is thus critical for understanding root functions and for promoting plant growth and crop productivity [2, 4].

Unlike the above-ground part of the plant, the poor accessibility of roots makes them a much more challenging target for phenotyping. The traditional approach of manually measuring root traits after excavation and root washing suffers from long processing times, possible human errors, and a limited set of traits that can be measured by hand. To improve efficiency, as well as the objectivity and veracity of root traits, modern phenotyping methods have resorted to imaging and computational processing. Earlier methods often rely on two-dimensional images of roots [5–8]. While such images are usually easy to obtain, these methods cannot fully capture the 3D shape and organization of roots [9]. As a result, a growing body of research utilizes 3D imaging technologies, such as X-ray CT, MRI, and multi-view optical imaging, to more accurately quantify the RSA [10–13].

Computational analysis of root traits from 3D imaging is a non-trivial task, due to the complex branching structure of a root system and the inherent ambiguity in the image data [9, 14]. Methods analyzing 3D root images commonly produce overall traits such as volume, depth, total root length and number [15–18]. These coarse-grained traits, however, do not capture the detailed organization and geometry of individual roots. More recently, several software packages have emerged that are capable of analyzing fine-grained root traits using X-ray CT imaging [19, 20] and optical imaging [21–23]. These methods can identify individual roots, measure their geometry (e.g., diameter, length, angle, tortuosity), and recover their spatial and hierarchical organization (e.g., junctions and lateral root orders). Some of these methods, such as DynamicRoots [21] and 4DRoot [20], can further produce dynamic growth traits by registering multiple samples across time.

**Fig. 1:**
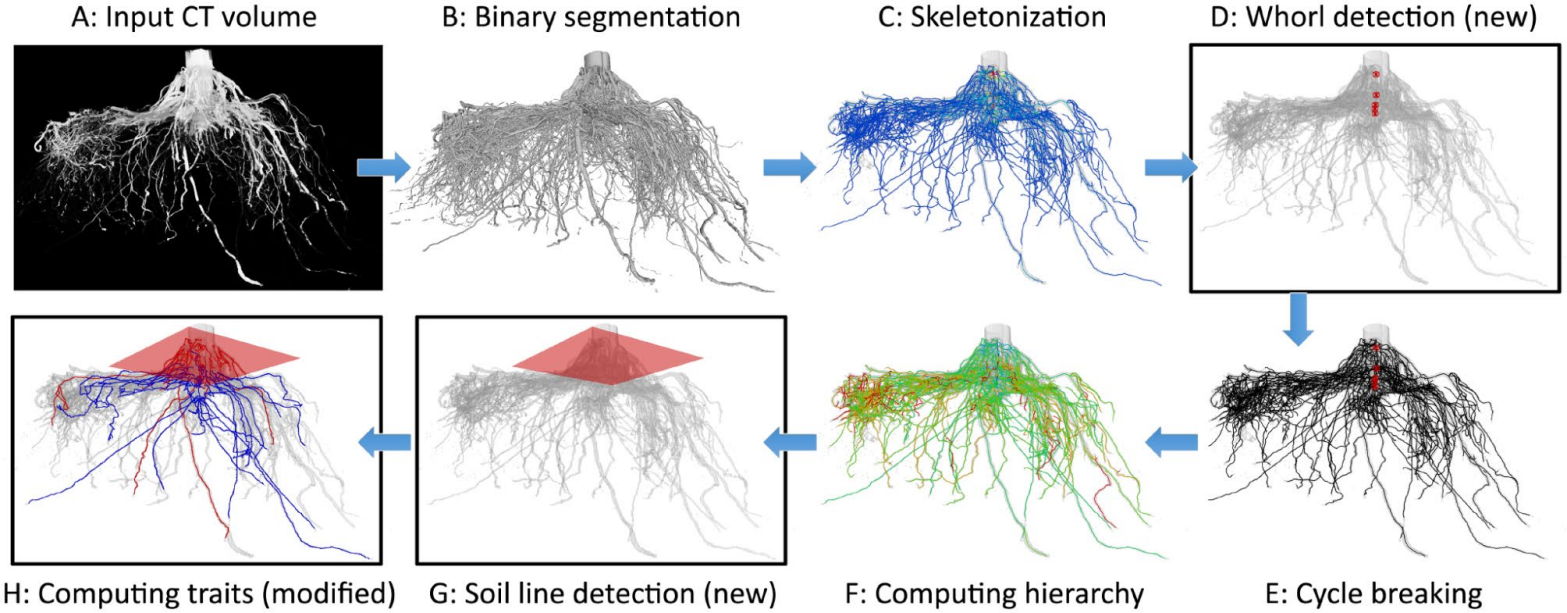
The TopoRoot+ pipeline for phenotyping excavated maize root crowns from CT images. Given an input 3D image (**A**), the pipeline computes a binary segmentation (**B**), extracts a curve skeleton (**C**, colored by thickness), detects whorls on the root stem (**D**), removes cycles on the skeleton (**E**), computes the hierarchical labels of skeleton branches (**F**, colored by labels), detects the soil line (**G**, red plane), and finally computes root traits (**H**, above- and below-ground nodal roots are colored in red and blue). The boxed steps are either new in TopoRoot+ or modified from the original TopoRoot pipeline

For nodal root systems (e.g., maize), a notable missing component in the traits computed by existing fine-grained phenotyping methods is the identification of *whorls*. Whorls are discrete locations at the base of each stem node where so-called “nodal” roots emerge circumferentially [22]. The number and location of whorls, and their associated nodal roots, are important traits for understanding genetic and functional variation of root system architecture [24]. However, identifying the precise locations of whorls from 3D images is far from a trivial task. First, as roots often cling to the stem, and due to the limited resolution of imaging, it can be difficult to locate the starting point of each nodal root (see Fig. 1A). Second, as the roots become denser around older (and deeper) whorls, these whorls are harder to detect. The only method we are aware of that produces whorl traits is DIRT/3D [22]. This method tracks the emergence of nodal roots from the top of the stem using a level-set method. However, as this method is designed for optical imaging, the occlusion of the roots prevents the method from reliably detecting whorls beyond the top few (usually 2).

A related root trait, also missing from existing works, is the location of the soil line on field-excavated root crowns. The soil line separates the above-ground portion of the root stem from the below-ground part, and it enables the computation of traits specific to each portion. This is important because while both above- and below-ground roots make contributions to plant architecture, only below-ground roots make contributions to water and nutrient absorption. Furthermore, this distinction allows for explicit quantification of root length density (the length of roots per volume of soil below-ground), which is considered to be one of the most valuable root functional traits. However, the location of soil line is far from being obvious after the roots are excavated, washed, and imaged.

In this paper, we propose a new method for identifying whorls and soil lines in X-ray CT images of field-excavated maize crowns, and for computing related fine-grained root traits. Our method, called TopoRoot+, is built on the recent TopoRoot pipeline [19]. TopoRoot employs topological simplification and skeletonization to produce a complete hierarchy of roots from CT images of maize crowns. TopoRoot + augments TopoRoot by adding two new modules, one for detecting locations of whorls and their associated nodal roots, and another for computing the soil lines, both via the analysis of the skeleton representation of the root crown. These new modules not only enrich the root hierarchy with whorls and the soil line position, but also add new traits including internode distances, root counts, geometry, and hierarchy levels at each whorl, and aggregated traits for the below-ground or above-ground part of the root crown.

We validated a subset of the new traits computed by TopoRoot+ (internode distances, nodal root count by whorls, soil line location and above/below-ground nodal root counts) against manual measurements on 133 field-excavated maize root crowns imaged using X-ray CT. The samples include nine genotypes and were grown in three separate years. Our experiments showed that the internode distances computed by TopoRoot + achieved an 11.6% error for the youngest internode distance and 15.5% cumulative error for all internode distances. The computed nodal root counts have 84.3%, 75.6% and 73.0% correlation at the 1st, 2nd and 3rd youngest whorls. Finally, the computed soil line locations are 92% accurate within 1 cm and 72% accurate within 5 mm from the actual soil line.

The TopoRoot + pipeline runs within minutes on a typical volume of size $\:40{0}^{3}$ on a standard desktop server, adding just seconds of extra time on top of TopoRoot. The efficiency of TopoRoot + makes it ideal for batch processing of excavated maize root images in a high-throughput analysis pipeline. The software is freely distributed on GitHub with the complete set of test data used in this paper. The distribution also includes an interactive graphical interface for viewing and editing root hierarchy, whorls, and soil lines.

## Methods

### Overview

Our method is built upon TopoRoot [19], a phenotyping pipeline designed for field-grown maize root crowns. Given an X-ray CT scan of an excavated root crown after root washing, TopoRoot produces a root hierarchy and fine-grained traits. However, TopoRoot does not detect whorls or the soil line, and hence it does not produce traits specific to whorls (e.g., internode distances, number of nodal roots per whorl, etc.) or the soil line (e.g., number of above- and below-ground nodal roots). This deficiency is addressed by our new work.

**Fig. 2:**
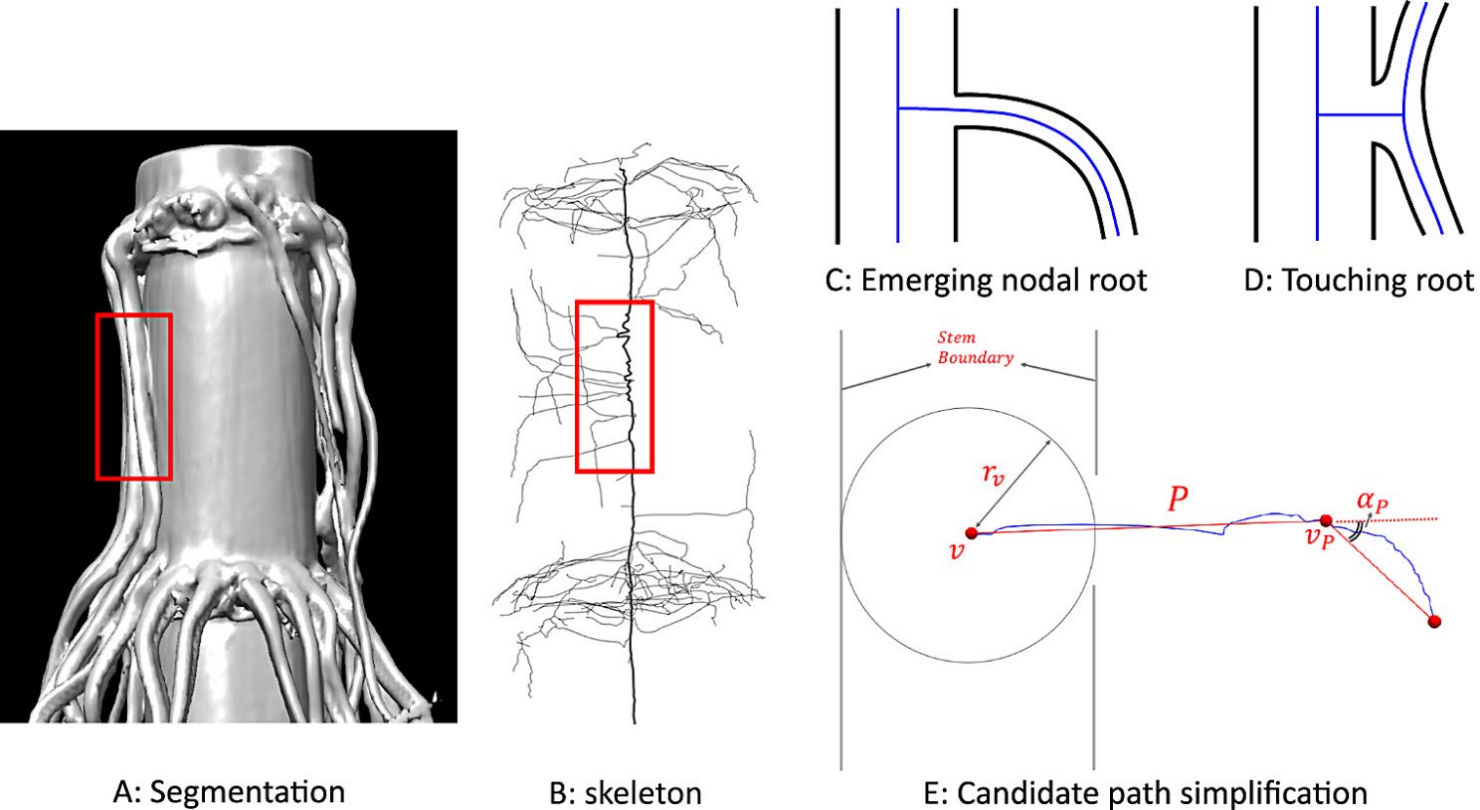
Left: nodal roots clinging to the stem (**A**, highlighted in red box) result in spurious skeleton curves connected to the stem path (**B**, thick curve is stem path). Top right: schematic illustration of an emerging nodal root (**C**) and a nodal root touching the stem (**D**), showing the root shape (black) and skeleton (blue). Bottom right (**E**): an example of a candidate path (blue) and its simplified path (red) with symbols used in the scoring function

The original TopoRoot pipeline proceeds as follows. First, a binary segmentation of the root crown is computed from the input grayscale volume (Fig. 2B). This segmentation maximally removes topological noises, such as islands, cavities, and handles, that arise due to the limited resolution and contrast of CT imaging. Second, a curve skeleton that captures the root branches, equipped with the thickness of a branch at each skeleton vertex, is computed from the segmentation (Fig. 2C, colored by thickness). The skeleton captures both the connectivity and geometry of individual roots. Third, cycles on the skeleton (as a result of the remaining topological noise in the binary segmentation) are removed using a minimal spanning tree (Fig. 2E). Fourth, the hierarchy labels (e.g., 0 for stem, 1 for nodal roots, 2 for first-order lateral roots, etc.) are computed for each skeleton branch (Fig. 2F, colored by hierarchy label). Finally, a suite of root traits is computed based on the hierarchical labels and the geometry of the skeleton branches. These traits include both aggregate measures, such as total root number and root length, and fine-grained measures, such as the number, length, tortuosity, and angles of roots at each hierarchy level.

We augment the TopoRoot pipeline with two new steps, one for detecting whorls (Fig. 2D) and another for detecting the soil line (Fig. 2G). Specifically, the whorl-detection step succeeds the skeletonization step and leverages the skeleton structure to identify clusters of nodal roots that emerge from the stem. The soil-line-detection step takes place after the computation of hierarchy and relies on analyzing the density of lateral roots (with label greater than 1). We also modified the trait-computation step to compute additional traits pertaining to whorls and the soil line (Fig. 2H). The augmented pipeline, which we call *TopoRoot+*, is illustrated in Fig. 2. Next, we detail the new and modified steps in their order in the pipeline.

### Whorl detection

The skeletonization step in TopoRoot produces a skeleton of the root crown that captures individual roots as skeleton curves. It also identifies the skeleton curve representing the root stem, called the *stem path*, by analyzing the thickness measures associated with the skeleton vertices (based on the observation that the maize root stem is usually thicker than the nodal or lateral roots). Ideally, each skeleton curve that branches off the stem path should represent a nodal root, and a collection of such curves whose branching points are nearby on the stem path should correspond to a whorl. In reality, however, not all skeleton curves that emerge from the stem path represent nodal roots. This happens, for example, when nodal roots (particularly those above the ground) have a steep growth angle toward the gravity vector and “cling” to the stem (see Fig. 1A), resulting in spurious skeleton curves connecting the nodal roots with the stem (see Fig. 1B). Drying during sample preparation can exacerbate this phenomenon. If left untreated, these spurious connections on the stem path would result in false-positive detection of whorls that do not exist.

We develop a method to distinguish between skeleton curves branching off the stem path that represent nodal roots from those that do not. The key observation is that a skeleton curve representing a nodal root usually bends smoothly under gravity (Fig. 1C), whereas a skeleton curve representing a false connection tends to bend sharply near the boundary of the stem, in order to join the skeleton curve that represents the nodal root clinging to the stem (Fig. 1D). In the following, we use this observation to score each skeleton curve’s likelihood of representing a nodal root.

We consider a skeleton vertex as *inside the stem* if its distance to the nearest (in terms of Euclidean metric) vertex $\:v$ on the stem path is no more than 1.2 times the thickness measure at $\:v$, denoted by $\:{r}_{v}$. Recall this measure, which captures the thickness of the branch in which the skeleton vertex lies, was available after the skeletonization step of TopoRoot (Fig. 2C). A skeleton edge is considered on the *stem boundary* if one of its two vertices is inside the stem and the other is not. We call a skeleton curve a *candidate path* if it is the shortest path on the skeleton that connects a vertex on the stem path with a skeleton edge on the stem boundary.

Candidate paths are found by employing the classical Dijkstra’s algorithm [25]. Given an edge-weighted undirected graph $\:G$ and a vertex $\:v$ in $\:G$, this algorithm finds the shortest paths, where the length is measured by the sum of edge weights on the path, from $\:v$ to every other vertex in $\:G$. Starting from $\:v$, the algorithm constructs a tree of shortest paths (known as the *shortest-path tree)* by iteratively adding a vertex with the currently shortest path from $\:v$. In our method, for each junction vertex $\:v$ on the stem path, we use Dijkstra’s algorithm to compute the shortest paths from $\:v$ to all skeleton vertices inside the stem that are incident to some skeleton edge on the stem boundary (whenever such path exists). Here, the weight of a skeleton edge is its length. We consider edges on the stem path, as well as edges whose vertices are both outside the stem, to have infinite weight to avoid paths using those edges. Each found shortest path becomes a candidate path after adding the skeleton edge on the stem boundary that is incident to the last vertex of the path.

Note that a candidate path may have a jagged geometry due to imaging noise and the skeletonization process, which could have a significant impact on the score. As a result, we first simplify the path using the Ramer-Douglas-Peucker (RDP) algorithm. Given an input polyline $\:P$ and a distance threshold $\:ϵ$, the RDP algorithm uses a greedy heuristic to find a polyline with the least number of vertices whose maximum distance to $\:P$ is no more than $\:ϵ$. Starting from a straight segment connecting the two ends of $\:P$, the heuristic finds the point $\:v$ of $\:P$ furthest to the segment. If the distance from $\:v$ to the segment is above $\:ϵ$, $\:v$ is inserted into the segment to form a better approximation of $\:P$. The point $\:v$ divides $\:P$ into two shorter polylines, and the heuristic proceeds recursively on each. When applying RDP to simplify a candidate path, we always perform the first step of the algorithm (adding the furthest point in $\:P$ to the straight segment connecting the two ends of $\:P$) to ensure that the simplified path contains at least three vertices. This is required for angle measurement (see below). Based on our empirical observation, we set $\:ϵ$ to be the size of 3 voxels in the input image volumes, which is equivalent to 1.32 mm in physical space. Examples of candidate path simplification are shown in Fig. 1E.

Now consider a simplified candidate path $\:P$ that starts at some stem path vertex $\:v$. We call the vertex of $\:P$ with the largest turning angle the *turning point* of $\:P$, denoted by $\:{v}_{P}$, and denote its turning angle (in radians) as $\:{\alpha\:}_{P}$ (see illustration in Fig. 1E). The score of $\:P$ is defined as,

$$\:S\left(P\right)=exp(-\frac{2{\alpha\:}_{P}}{\pi\:}-\frac{10\:-min\left(max\right(|v-{v}_{P}|-{r}_{v},\:0),\:10)}{10})$$

This score is closer to 1 for smaller turning angles $\:{\alpha\:}_{P}$ and greater distances from the turning point $\:{v}_{P}$ to the stem boundary, which is measured by $\:\left(\right|v-{v}_{P}|-{r}_{v})$ and clamped to be within the range from 0 to 10 voxels (equivalent to 4.4 mm). This scoring function captures the observation earlier that a nodal root tends to bend less sharply, and the bending location is further away from the stem, than a false connection on the stem.

Note that our candidate path detection algorithm above may produce multiple candidate paths connecting the same skeleton edge on the stem boundary to different junction vertices on the stem path. After computing the scores, and for each skeleton edge on the stem boundary, we keep only the candidate path with the highest score containing that edge.

**Fig. 3:**
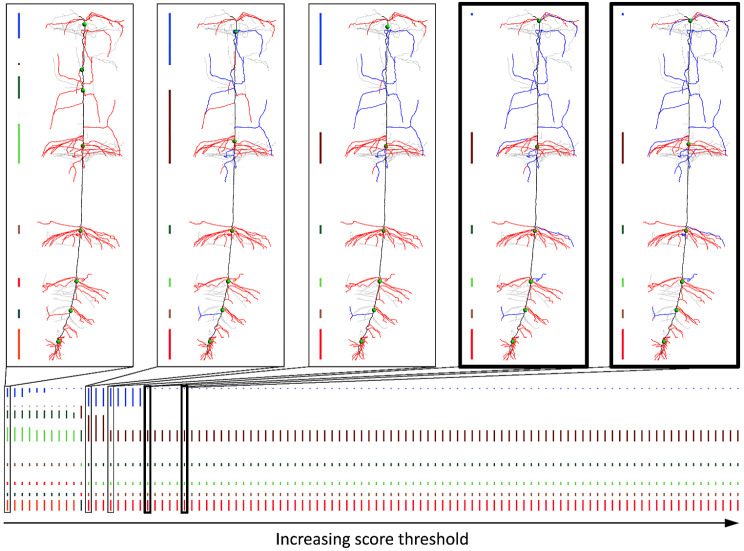
Top: Clustering of candidate paths on the skeleton at several score thresholds. Paths above and below the thresholds are colored red and blue (gray skeleton curves are not candidate paths). Each cluster is depicted by a bar on the left, whose length is proportional to the distance (along the stem) between the highest and lowest candidate paths in that cluster. Green balls mark the whorl locations. Bottom: The bar visualization of clustering results for all score thresholds in increasing order. The thickened boxes in both top and bottom highlight the same clustering result, which persists for the most thresholds

A straight-forward way to detect whorls using the scoring function above is to cluster all candidate paths whose scores are above some threshold $\:\delta\:$. However, the clustering result can be sensitive to the choice of $\:\delta\:$, as shown in Fig. 3 top. To avoid the need for manually tuning $\:\delta\:$, we observed that the desirable clustering is usually quite stable with respect to small changes in $\:\delta\:$. Following the observation, we adopt a parameter-free approach, which seeks the clustering result that remains the same for the *longest range* of thresholds.

Specifically, let $\:\varDelta\:$ be the list of scores for all candidate paths, sorted in ascending order. For each value $\:\delta\:$ in $\:\varDelta\:$, we consider all candidate paths whose scores are no smaller than $\:\delta\:$, and cluster the locations of their starting vertices along the stem path. We used the mean shift algorithm [26], which is a well-known non-parametric clustering technique that is well-suited for scenario where the number of clusters is unknown. Two clustering results are considered the same if (1) they consist of the same number of clusters and, if so, (2) each pair of corresponding clusters share the same highest and lowest candidate paths. Figure 3 bottom visualizes the clustering results for all values in $\:\varDelta\:$ for one root sample. We then take the clustering that remains the same for the most consecutive values in $\:\varDelta\:$ as the final clustering (the last two clustering results in Fig. 3 top). Each cluster in this clustering becomes a whorl. Since false connections are rare near a true whorl, we consider all candidate paths (regardless of their scores) between the highest and lowest candidate path of each whorl as representing nodal roots of the whorl.

Following whorl detection, TopoRoot + continues with the cycle-breaking step of TopoRoot (Fig. 2E). We have observed that false connections on the stem with clinging nodal roots often result in cycles in the skeleton graph. To improve the accuracy of the cycle-breaking step of TopoRoot, we added a final step in whorl detection to remove cycles resulted from false connections. Specifically, we trace paths of skeleton edges that originate from the stem path but do not belong to any candidate paths representing nodal roots. The traced paths are either not candidate paths, or candidate paths with low scores that are not included in the clustering. The tracing of a path starts from a junction vertex on the stem path and stops at the next junction vertex. After tracing, all vertices (except the two at the ends) and edges on the traced paths are removed from the skeleton.

### Soil line detection

**Fig. 4:**
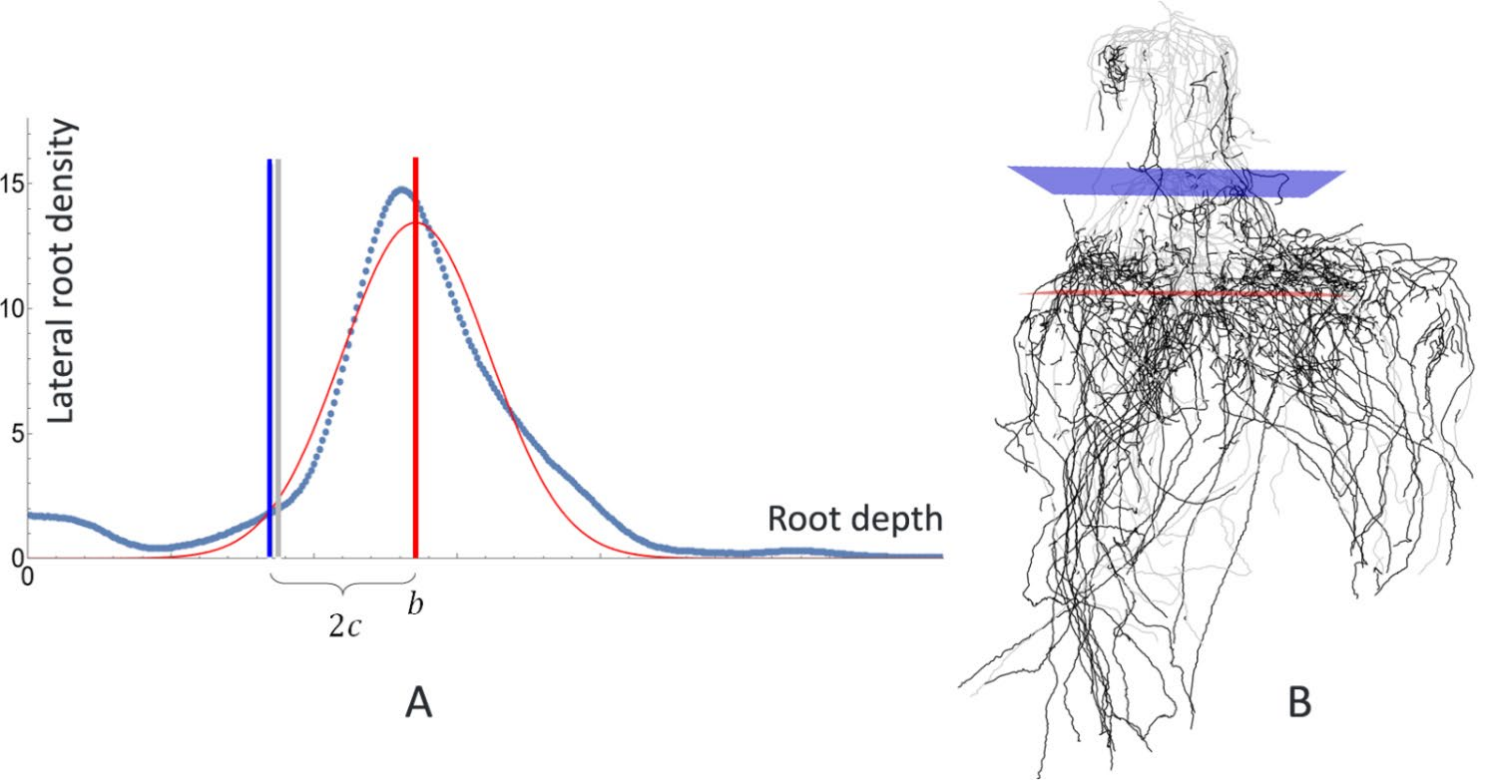
**A**: Lateral root density as a function of the root depth (dark blue dots) on one root crown, the best-fit Gaussian (red curve; $\:b$ and $\:c$ are mean and standard deviation), and the locations of the pinhole (gray line) and the computed soil line (blue line). **B**: Visualizing the skeleton (lateral roots are black), the peak of lateral root density (red plane), and the computed soil line (blue plane)

While soil lines are easily identifiable when imaging plant roots in vivo, they are generally not visible on excavated and washed maize roots. Our technique is based on the observation that for excavated maize root crowns, the density of lateral roots is significantly higher below the ground than above the ground. To verify this observation, and to study the relation between soil line location and lateral root density, we conducted an experiment where we inserted pushpins into the root stem at the soil level before excavating the root crowns. The pins were removed before CT imaging, which left holes in the stem that can be manually identified in the 3D images. We then process the images using the TopoRoot pipeline (with whorl detection) to obtain the root hierarchy, and plot the density of lateral root (of any order) as a function of vertical depth. We observed that this density function often has a Gaussian-like shape, and the location of the pinhole on the stem roughly lies where the function rises (Fig. 4A, B).

Guided by the observation, we detect the soil lines by first fitting the density function of lateral roots with a Gaussian function, $\:f\left(x\right)=aexp(-\frac{(x-b{)}^{2}}{2{\:c}^{2\:}})$, where $\:a,b,c$ are the height of the peak, location of the peak (measured top-down), and the standard deviation. The soil location on the stem is then determined by $\:b-2\:c$. The factor of 2 is chosen based on our observation of the pinhole data (Fig. 4A, B; see validations in the Results section). While our method is designed for field-excavated maize, the principle behind our method is likely applicable to other plants with proliferation of lateral roots after nodal root soil penetration.

### Computing traits

**Fig. 5:**
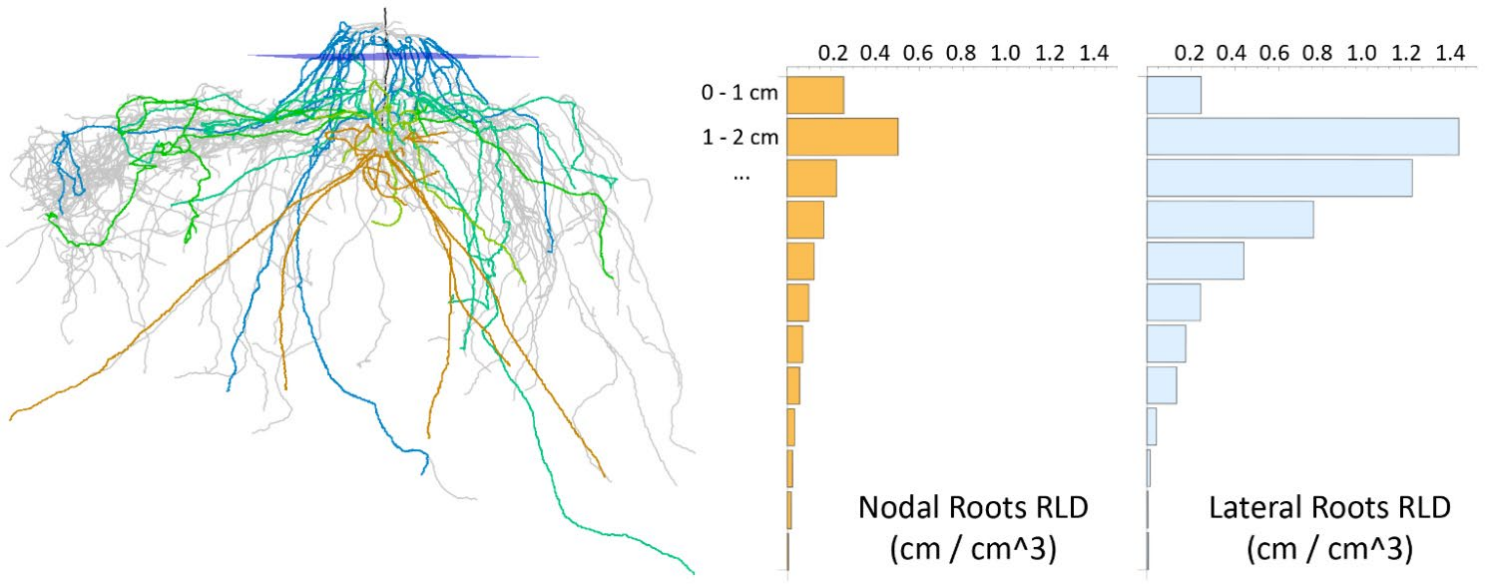
Root length density (RLD, right) of nodal roots and lateral roots in a root crown (left) plotted as bar graphs, where each bar represents the total length of roots (in cm) per unit soil volume (in cubic cm) for each centimeter of depth under the soil line

With the detected whorls and soil line, we can augment the root traits produced by TopoRoot with a suite of new traits, including:

1. Total whorl number, and internode distances between every two consecutive whorls. The location of each whorl is the mean of the starting locations of all candidate paths representing the nodal roots of that whorl (see details in “*Whorl Detection*”). The internode distance between two whorls is measured as their distance along the root stem.
2. For each whorl and each hierarchy level, the root count, total and average root lengths, average root tortuosity, average root thickness, average number of children, and the average emergence, midpoint, and tip angle.
3. Traits in (2) aggregated for all above-ground whorls and all below-ground whorls.
4. Root length density (RLD) computed as the total length of nodal roots and lateral roots per unit soil volume for each centimeter depth under the soil line (see an example in Fig. 5). We consider all roots in a “virtual” soil core - a cylinder whose axis is aligned with the root stem and whose radius covers 95% of all roots in our data set (which is 8.60 cm).

The complete list of all traits can be found in Supplementary Table 1.

## Results

We validated a subset of the traits computed by TopoRoot + against manual measurements field-excavated root crowns. These traits include internode distances, nodal root count per whorl, soil line location, and above-ground and below-ground nodal root count. Our pipeline was implemented in C++, and all experiments were performed on a Windows 10 machine with an Intel(R) Core(TM) i9-10900X Processor @ 3.70 GHz and 64.0 GB of memory (RAM).

### Data preparation

To evaluate the algorithm across a range of real-world phenotypic variation that we encounter in maize field experiments, we constructed a data set that features multiple site-years and numerous genotypes. Three cohorts of maize seeds were planted in June of 2020 and 2021 in O’Fallon, MO (for 2020 and 2021) and in 2022 in St. Charles, MO (for 2022). The 2020 and 2021 cohorts consist of a mutant (Rt1-2.4 MUT) with a mutation in the *Rootless1* gene, which was known to alter nodal root patterning [27], and its fully functional wildtype (maize inbred genotype T43). The 2022 cohort consists of a small genetic diversity panel (maize inbreds Ki11, CML228, CML247, CML333, Tx303, NC350, and B73) from the parental lines of the maize Nested Association Mapping panel (NAM Founders).

**Fig. 6:**
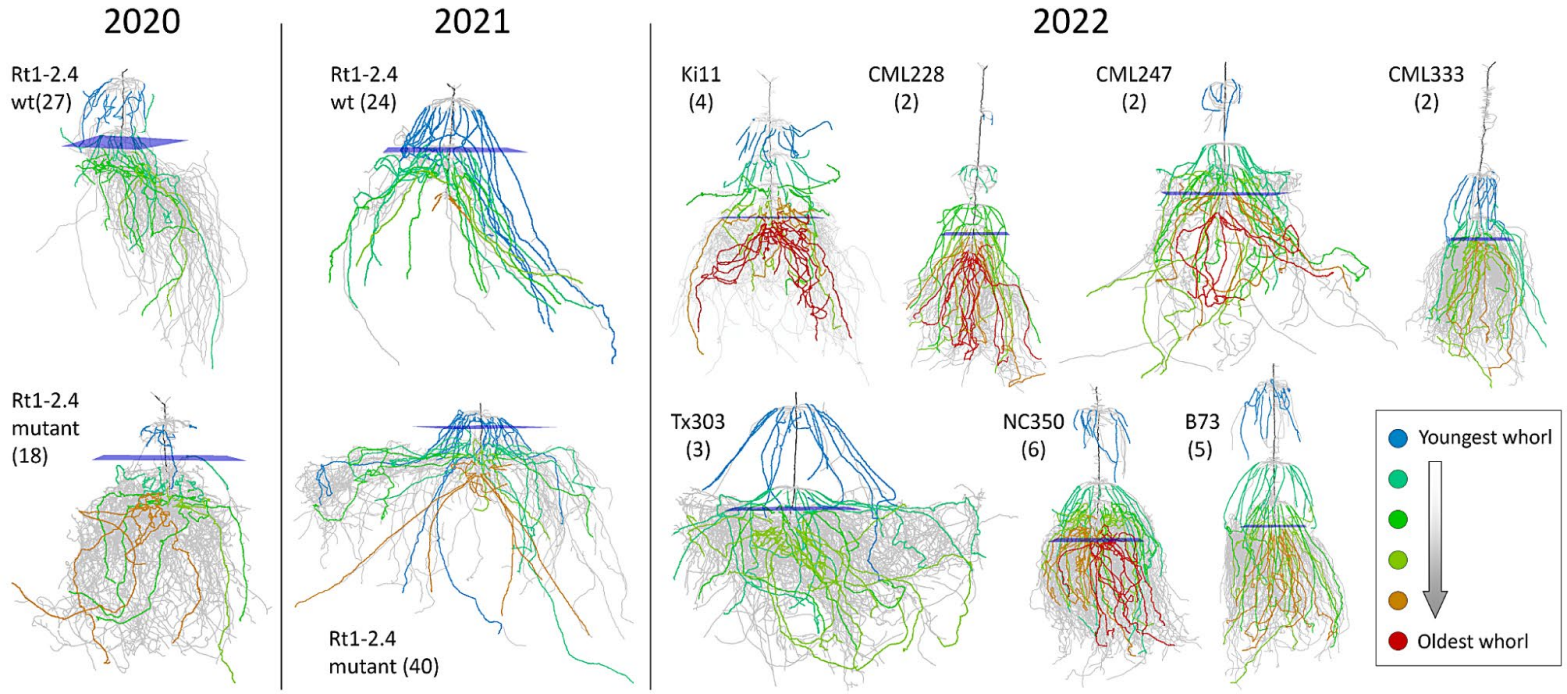
Result of TopoRoot + on selected root samples from the 2020, 2021 and 2022 cohorts. One sample is shown for each genotype in each cohort, and the number of samples of that genotype in the respective cohort is indicated in parentheses. Each result is shown as a skeleton where the nodal roots are colored by their whorls (see legend). The blue planes indicate the computed soil line locations

The seeds were planted in silt loam soil using jab-type planters, and genotypes were planted in single rows with a complete randomized design. Roots were excavated after 54–57 days of growth (for 2020 and 2021 cohorts) or at anthesis (for 2022 cohort) using the Shovelomics protocol [28] and washed to remove large chunks of soil. X-ray computed tomography (XRT) was conducted using an X5000 X-ray imaging system, with the X-ray source set to a voltage of 70 kV, current of 1700µA, and focal spot length of 119 μm. Each sample was clamped and placed on a turntable for imaging at a magnification of 1.17X and 10 frames per second, resulting in 1800 16-bit digital radiographs over a 3-minute scan time. The radiographs were reconstructed into a 3D volume at 109 μm voxel resolution using the efX-CT software, which was then exported as a 16-bit RAW volume. Following TopoRoot, we down-sampled each volume by a factor of 4 in each dimension for efficient processing. After removing volumes with excessive soil present, we obtained 45, 64, and 24 3D root volumes respectively in the 2020, 2021, and 2022 cohorts for this validation study. Note that the 45 samples from 2020 were identical to those used for validating TopoRoot [19]. Figure 6 shows the result of our pipeline (as skeletons) for selected examples from each cohort.

For validation purposes, we obtained manual measurements of internodal distances and nodal root count per whorl for all three cohorts. Each sample was dissected starting from the highest whorl (stalk end) and moving downward. Nodal root counts considered only attached roots at each whorl. Furthermore, as mentioned earlier, and for validating our soil line detection algorithm, a pushpin was inserted in the root stem of each sample in the 2021 and 2022 cohorts at the soil level, either prior to root excavation (in 2021) or after excavation and based on visual examination of the pigmentation and lateral root branching (in 2022). The pushpins were removed before CT imaging. The pinhole in the root stem left by the pushpin can be unambiguously identified in the 3D volume for all 64 samples in the 2021 cohort but only 14 samples in the 2022 cohort. For each of these 78 samples, we manually recorded the location of the pinhole in the Z direction of the volume, and classified the manually found whorls to be either above or below the ground. Note that these manual evaluations are somewhat subjective and prone to error, especially as the nodes get smaller and whorls more tightly appressed near the seed.

### Whorl traits

We first measure the deviations between the internode distances computed by our algorithm and those measured by hand. Let $\:{c}_{i}$ and $\:{m}_{i}$ denote respectively the computed and measured distance between the $\:i$-th and $\:(i+1)$-th node, where the index starts from the youngest whorl ($\:i=1$). We define the following cumulative error of the first $\:k$ internode distances (for $\:k\ge\:1$):

$$\:{E}_{k}=\frac{{\sum\:}_{i=1}^{k}\left|\right|{c}_{i}-{m}_{i}\left|\right|}{{\sum\:}_{i=1}^{k}{m}_{i}}$$

**Fig. 7:**
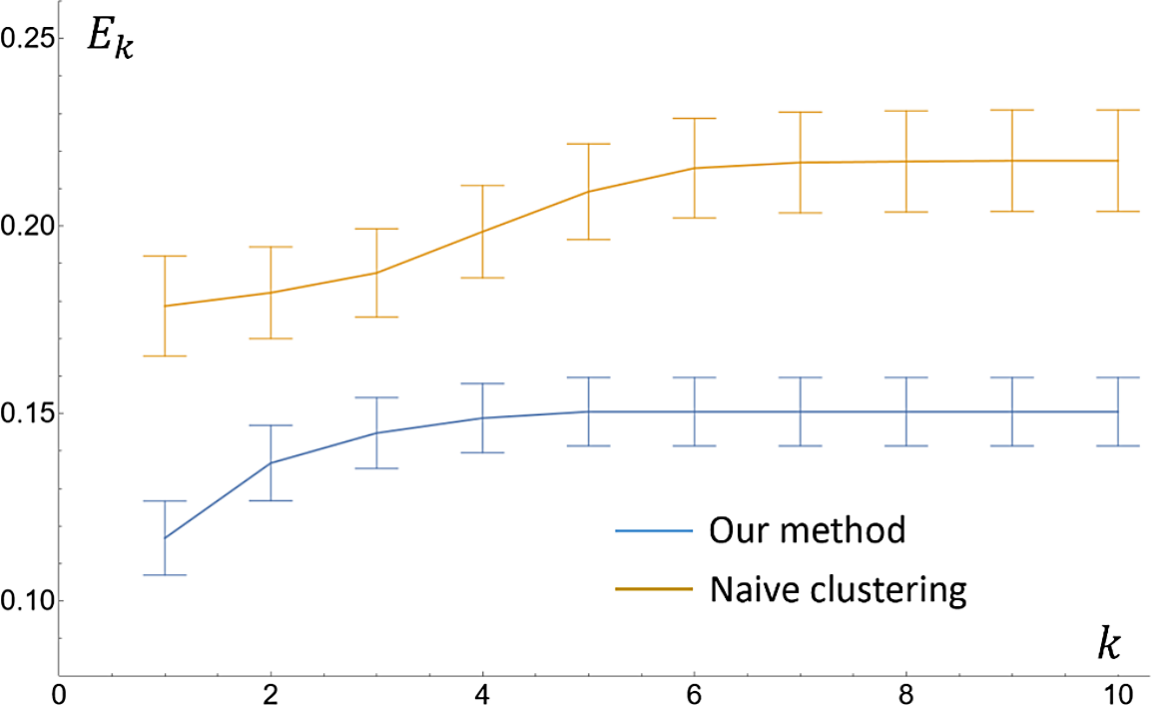
Accumulated error (compared with manual measurements) of the first $\:k$ internode distances, $\:{E}_{k}$, as functions of $\:k$ for all samples from the three cohorts (2020, 2021, 2022)

The mean and standard deviations of $\:{E}_{k}$, for all samples in the three cohort (2020,2021,2022), are plotted as functions of $\:k$ in Fig. 7 (see blue curves and error bars). Observe that the error is lowest for the first internode distance (between 9 and 13%) and rises as lower (and older) whorls are considered. This rise is due in part to the increase in root density around older whorls, which makes accurate detection of whorls more challenging (see Discussions). In addition, missing or redundant whorls would change the matching between subsequent detected and measured whorls, and the impact of such mismatch on the error increases with $\:k$. The error plateaus (between 13 and 16%) towards the bottom of the root stem, since the last few internode distances are typically very small and hence contribute little to the accumulated error. Considering samples in all three cohorts, the average error in the first internode distance, $\:{E}_{1}$, is 11.6% with a standard deviation of 1.0%, and the average overall error, $\:{E}_{k}$ where $\:k$ is the total number of measured whorls in each sample minus one, is 15.5% with a standard deviation of 0.9%.

To further justify our algorithmic choices, Fig. 7 shows the error of a naive whorl detection method (orange curves and error bars), which simply performs mean-shift clustering of all candidate paths on the root stem (without scoring). Compared with our algorithm, which performs adaptive clustering after scoring the candidate paths, the naive algorithm yields a much higher error range (starting at 15% and plateauing at 21%). There is a significant difference between the errors $\:{E}_{k}$ of our method and of the naïve method at all $\:k$ (the maximum p-value is 0.0082, at $\:k=3$). The higher errors are mostly caused by the spurious skeleton branches that correspond to nodal roots touching the stem (see Fig. 1).

The curves and error bars are the mean and standard deviation. Errors produced by our algorithm are in blue, and errors produced by a naive clustering algorithm of candidate paths (without scoring) are in orange.

**Fig. 8:**
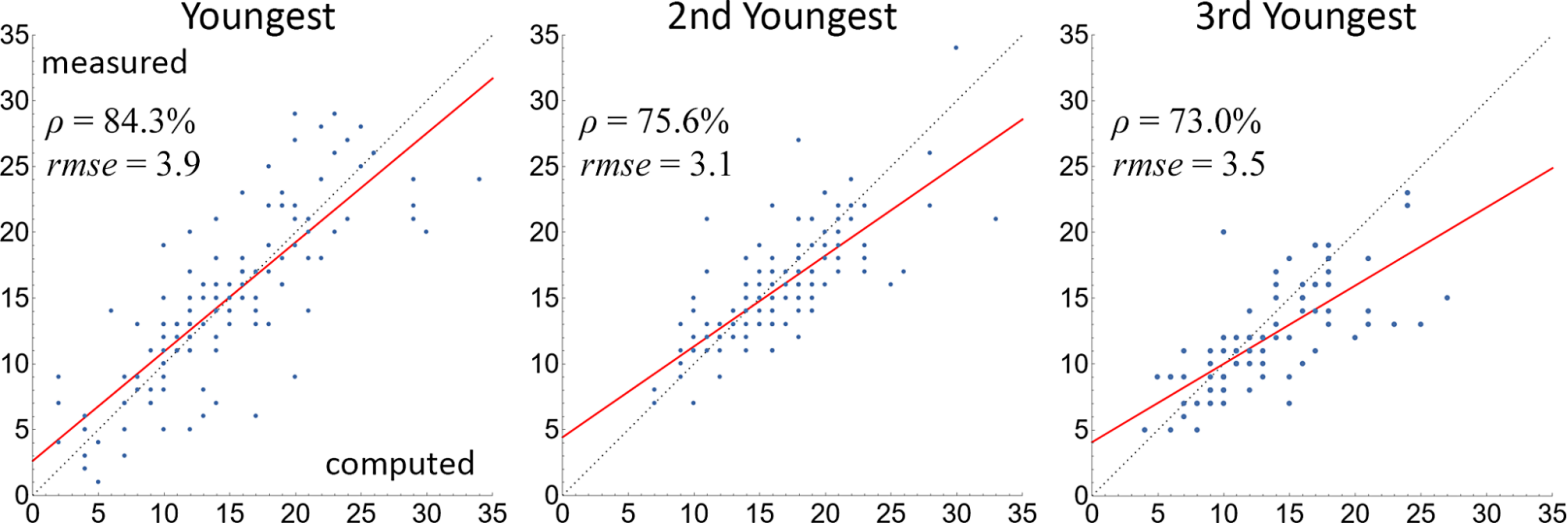
Scatter plots of nodal root counts at the top (youngest) three whorls for all samples in all three cohorts (2020, 2021, 2022). The horizontal and vertical axes are the computed and measured counts, and each dot represents one sample. The dashed gray lines indicate a perfect match between the computed and measured counts, and the regression lines are colored red. The Pearson correlation coefficient ρ and RMSE between computed and measured counts are indicated in each plot

We next examine the nodal root count at each whorl. Figure 8 visualizes the measured and computed nodal root counts for the top three whorls for all samples from three cohorts. At the youngest whorl, the Pearson correlation coefficient between the computed and the measured counts is 84.3%. The correlation drops at the second and third youngest whorls, becoming 75.6% and 73.0%. As in the internode distance validation, we attribute the decreased correlation at older whorls to the increased difficulty in identifying older whorls and the cumulative effect from missing or redundant whorls.

### Soil line traits

**Fig. 9:**
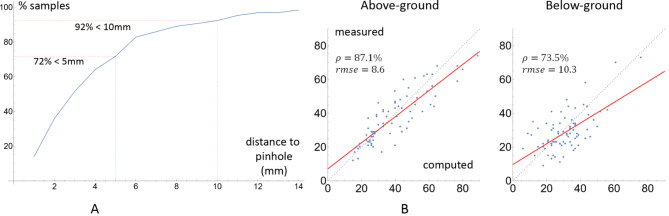
**A**: The graph of the percentage of 78 samples in the 2021 and 2022 cohort where the computed soil line position is within a given distance from the pinholes, as a function of the distance. **B**: Scatter plots of total nodal root counts above or below the ground for all samples. The horizontal and vertical axes are the computed and measured counts, and each dot represents one sample. The dashed gray lines indicate a perfect match between the computed and measured counts, and the regression lines are colored red. The Pearson Correlation value ρ and RMSE for each scatter plot is indicated

To measure the accuracy of the soil line location computed by our algorithm, we consider its distance in the Z direction of the volume from the manually identified pinhole location on the root stem. As mentioned earlier, the pinhole locations are visible in 78 samples, including 64 in the 2021 cohort and 14 in the 2022 cohort. We plotted the percentage of all samples within a given distance from the pinhole, as functions of that distance, in Fig. 9A. We found that 72% and 92% of all samples are within 5 mm and 10 mm from the pinholes, respectively, indicating that our algorithm can locate the soil line with high accuracy.

We next consider the total nodal root count above and below the ground. For each of the 78 samples, we summed up the measured nodal root counts for all whorls that are above or below the pinhole, and compared them with the numbers computed by TopoRoot + using the detected whorls and soil line locations. These numbers are visualized in scatter plots in Fig. 9B. Consistent with our observations above, the computed nodal root counts are more accurate for above-ground nodal roots (87.1% correlation and 8.6 RMSE) than for below-ground ones (73.5% correlation and 10.3 RMSE).

### Comparison between wild-type and Rt1-2.4 MUT

**Fig. 10:**
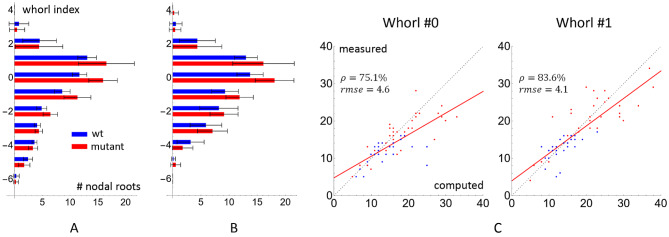
**A**: Comparing the average manual nodal root count at each whorl between wild-types (blue) and mutants (red) in the 2021 cohort. The whorls are indexed such as the 0-th whorl is directly above or at the soil line and younger whorls have higher indices. **B**: Same comparison as A but computed by TopoRoot + instead of measured by hand. C: Scatter plots of measured and computed nodal root counts at whorls 0 and 1, colored by genotypes (blue for wild-types and red for mutants)

As a further validation of our pipeline, we applied it to compare the two genotypes in the 2021 cohort, the wild-types (Rt1-2.4 WT) and mutants (Rt1-2.4 MUT). As shown in Fig. 10A the Rt1-2.4 mutation causes a different pattern of nodal roots at various whorls, most notably an increase in the number of roots at whorls close to the soil line (whorl 0 and whorl 1). Here, the whorls are indexed such that the 0-th whorl is the one directly above or at the soil line, and the indices decrease in the direction of gravity so that the remaining above-ground and below-ground whorls have positive and negative indices, respectively. The differences in nodal root count between the two genotypes are statistically significant at whorl 0 (*p* = 0.002) and 1 (*p* < 0.001).

We next compared the nodal root counts between the wild-types and mutants computed using TopoRoot+, as shown in Fig. 10B, and we observed a qualitatively similar pattern. In particular, the differences in nodal root count between the two genotypes are statistically significant at whorl 0 (*p* = 0.032) and 1 (*p* = 0.003). Scatter plots correlating the measured and computed nodal root counts at these two whorls, colored by the genotypes, are given in Fig. 10C. This validation demonstrates the potential of TopoRoot + in root studies involving whorls and soil line positions.

## Discussions

Despite the advance in imaging technology and its use in plant phenotyping, analysis of 3D root images remains a challenging task, particularly when it comes to extracting fine-grained architectural traits. This study advances the current literature of computational root phenotyping methods by offering a new suite of fine-grained traits that are related to whorls and soil lines of excavated root crowns. While these traits can only be obtained previously by tedious, destructive, and human-biased manual measurement, the automated TopoRoot + pipeline can potentially enable more biological studies concerning the whorl structure and/or specific to the above- or below-ground part of the root (we gave an example of comparing the nodal root count near the soil line between the wild-types and mutants). Although the proposed pipeline is designed for, and tested on, X-ray CT scans of field-excavated maize root crowns, the techniques are likely generalizable to other nodal root systems (e.g., sorghum with a single tiller) and imaging modalities (e.g., MRI). We next discuss our choice of parameters, the sources of errors of our method, its computational performance, and software availability, which includes a graphical and interactive tool for viewing and editing the root hierarchy.

### Parameters

There are several empirically determined parameters in our method, which are described in the Methods section: (1) the multiplier of 1.2 to the stem thickness to decide if a skeleton vertex is in the stem (in Whorl Detection), (2) the error threshold of $\:ϵ$ being 3 voxels in the RDP algorithm for curve simplification (in Whorl Detection), and (3) the multiplier of 2 on the standard deviation of lateral branching density in determining the soil line position (in Soil Line Detection). These parameters were estimated based on informal observations of a randomly selected subset of our data set. We did not fine-tune these parameters to maximize the accuracy of the entire dataset. It is possible that different parameters values might be needed for new datasets, depending on the species (which would affect the stem thickness multiplier), growing conditions (which might require a different multiplier in soil line detection), and imaging resolutions (which might call for a different value of $\:ϵ$).

### Error analysis

As TopoRoot + builds upon the TopoRoot pipeline [19], it also inherits the latter’s errors, which lie mostly in the binary segmentation and cycle-breaking steps (see Fig. 2). In particular, the segmentation may alter the structure of the root system (e.g., breaking up a root or merging disjoint roots), and the cycles may be broken in wrong locations (e.g., in the middle of a root). Note that our whorl detection algorithm can remove false skeleton curves that do not represent nodal roots, and hence reduce the connectivity errors on the skeleton.

Errors in whorl detection are most often caused by the scoring function of candidate paths on the skeleton. Our scores, which are based on the turning angle and turning point of the paths, are not always effective in distinguishing between paths representing nodal roots from those that do not. For example, paths representing nodal roots that bend sharply downward (often seen at whorls above the ground) may have low scores, while skeleton curves corresponding to multiple roots touching the root stem (often seen at whorls below the ground) may not have the characteristic shape shown in Fig. 1D and hence can have high scores. Although our proposed adaptive clustering method is more robust against scoring errors than straightforward clustering after applying a fixed threshold on the scores, missing or redundant clusters can still happen. A possible remedy is to incorporate additional geometric properties in the scoring function, such as the thickness of the root, which is also available on the skeleton. Furthermore, since whorls located close to the bottom of the root stem are typically closer to each other, they are often incorrectly merged in our clustering result.

Our soil line detection algorithm is based on a simple observation on the density of lateral roots in the vertical direction. We have identified several other visual cues about the location of soil lines. For example, the internode distances often sharply reduce below the soil, and so does the thickness of the root stem. Incorporating these cues into our current algorithm can potentially improve its accuracy in locating the soil line, which stands currently at 72% within 5 mm and 92% within 10 mm. Note that traits that require the detection of both whorls and soil line locations, such as total nodal root count above or below the soil, will be affected by errors in both detection algorithms.

As our work is focused on methodology development, we have not measured how the errors of the computed traits affect downstream biological studies. Such measurement, which we plan to conduct in the future in the context of specific studies, would allow us to further evaluate the practical value of our pipeline.

### Running time

The processing time of TopoRoot + is dominant by steps in the original TopoRoot pipeline, particularly topological simplification and skeletonization (Fig. 2B, C), both of which scale with the dimension of the input images. The additional steps introduced by TopoRoot, including whorl detection and soil line estimation, operate on the geometric skeletons. The time complexity of these steps scales with the size of the skeleton (e.g., number of vertices and edges), which is much smaller than the number of voxels in the image volumes. As a result, TopoRoot + introduces only a small overhead to the processing time of TopoRoot.

The CT volumes in the 2020 and 2021 cohorts have a similar size of roughly 400$\:\times\:$400$\:\times\:$400 voxels (after downsampling by a factor of 4 in each dimension). Processing each volume takes on average 13 min and 44 s, and only 21 s (2.5% of total time) on average are taken up by the new steps introduced in TopoRoot+. The 2022 cohort has a much larger variation in volume size, ranging from 246$\:\times\:$246$\:\times\:$310 voxels to 365$\:\times\:$365$\:\times\:$1120 voxels. The average running of TopoRoot + on one sample is 15 min and 48 s, of which only 19 s (2.0% of total time) is spent on whorl and soil line detection. The running time on the largest volume in the 2022 cohort is 32 min and 47 s.

Note that these running times are much shorter compared to those needed for preparing and imaging the root samples, and they can be batch-run on a computer server. As a result, the pipeline is well-suited for high-throughput processing of root cohorts. In the future, we will explore how to further accelerate the computations by parallelizing the pipeline on a multicore cluster.

### Software distribution

TopoRoot + is freely distributed online [29]. The GitHub page contains instructions to run the software, the complete set of root crown data used in our validation, and details on the input and output file formats. The program currently supports both image stacks and 3D volume formats, and it produces a skeleton, a hierarchy labeling of the skeleton, and a spreadsheet of root traits. Like TopoRoot, TopoRoot + currently only supports Windows 10 machines.

Also included in our distribution is a graphical interface for viewing and editing the root hierarchy and traits in 3D. The interface visualizes the segmented root system as well as its skeleton, colored based on various root features selected by the user (e.g., hierarchy level, whorl, above or below the ground, etc.). The user can selectively show or hide skeleton curves by hierarchy level or whorl, and see the whorl locations and soil planes. In addition, the user may alter the root hierarchy, whorls, or soil line interactively using mouse and keyboard controls (see Supplemental Fig. 1). More instructions can be found on the GitHub page.

## Conclusion

We introduced TopoRoot+, a high-throughput pipeline for fine-grained phenotyping of excavated maize root crowns imaged by X-ray CT. Building on top of the TopoRoot pipeline [19], the expanded work includes new algorithms for detecting whorls, the nodal roots at each whorl, and the soil line locations, thereby producing a suite of root traits that are not offered by existing computational methods. While many of these traits are not feasible to measure manually, we validated a hand-measurable subset of traits on three separate cohorts of field-excavated maize root crowns. Our pipeline is efficient to run and well-suited for high-throughput analysis. The program is freely distributed online and includes a visual interface for inspecting and editing the root hierarchy.

### Electronic supplementary material

Below is the link to the electronic supplementary material.

Supplementary Material 1

## Data Availability

The X-ray CT scans of all root crowns, along with hand measurements of nodal roots for each sample, are available in the TopoRoot + GitHub repository [29].
