# Supplementary material for: TopoRoot+: computing whorl and soil line traits of field-excavated maize roots from CT imaging
Source: Plant Methods. 2024 Aug 27;20:132. doi: 10.1186/s13007-024-01240-0 (PMC11348750; doi:10.1186/s13007-024-01240-0)
Supplement: Supplementary file 1 — Supplementary Material 1 [file 13007_2024_1240_MOESM1_ESM.docx]

Supplementary Information


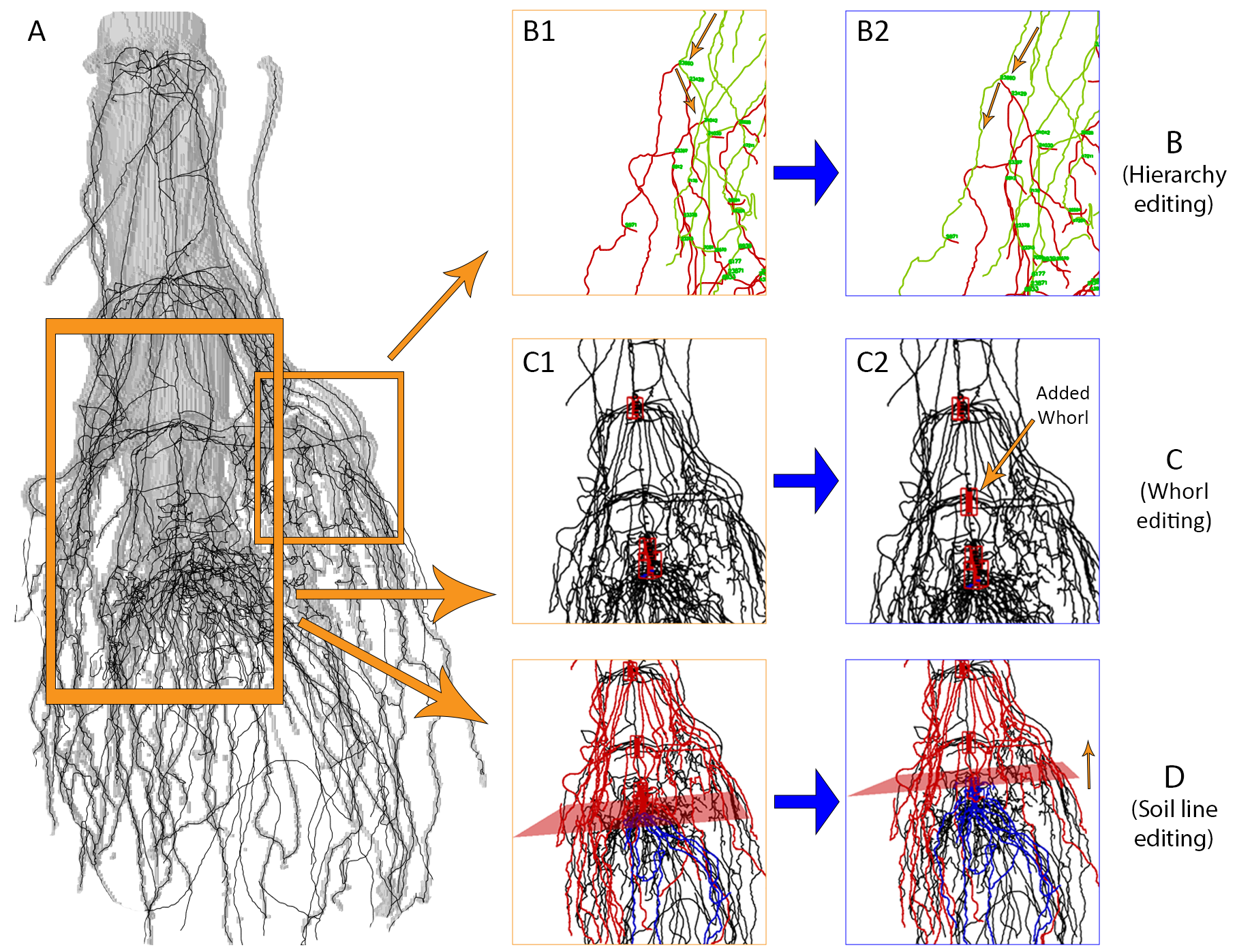
**Supplementary Figure 1**: Graphics User Interface (GUI) to view and edit root hierarchy, whorls and soil line. A: The segmented root shape (shown in transparency) and its skeleton. B: Fixing An incorrect root hierarchy by changing how the root continues at a junction (pointed by the top arrow in B1) from following the right branch to following the left branch (B2). C: Adding a missing whorl (in C1) by selecting a group of junctions on the root stem and creating a new whorl from them (C2). D: Shifting the soil plane upward.

**Supplementary Table 1: TopoRoot’s computed traits**

| **Groups** | **Trait** | **Description** |
| --- | --- | --- |
| Global traits | Total length | Sum of the lengths of stem, nodal roots, and lateral roots. |
|  | Number of roots | Total number of roots across all hierarchy levels. |
|  | Average length | Total length divided by number of roots. |
| Stem traits | Average stem thickness | Average thickness of the vertices along the stem path. |
|  | Stem length | Length of the stem path. |
| Per-level traits | Level *n* root count | The number of level *n* roots. |
|  | Total level *n* root length | Sum of the lengths of level *n* roots. The length is the skeleton distance from the beginning of the root to its tip. |
|  | Average level *n* root length | Total level *n* root length divided by level *n*  root count. |
|  | Level *n* root tortuosity | Length of a root (skeleton distance) divided by the Euclidean distance from the beginning to the tip, averaged across all level *n* roots. |
|  | Level *n* root thickness | Thickness associated with the skeleton vertices in the root, averaged across all level *n* roots. |
|  | Number of level *n* root children | Number of level 2 roots divided by number of level 1 roots. |
|  | Level *n* root tip angle | Angle between the stem direction and the vector from the beginning to the tip of a root, averaged across all level *n* roots. |
|  | Level *n* root emergence angle | Angle between the stem direction and the vector from the beginning to 30 vertices along the skeleton of a root, averaged across all level *n* roots. |
|  | Level *n* root midpoint angle | Angle between the stem direction and the vector from the beginning of a root to the halfway point of the root, averaged across all level *n* roots. |
| Aggregated lateral root traits | Total lateral length | Sum of the lengths of lateral roots whose hierarchy level is greater than or equal to 2. |
|  | Number of lateral roots | Number of lateral roots whose hierarchy level is greater than or equal to 2. |
|  | Average lateral root length | Average length of lateral roots whose hierarchy level is greater than or equal to 2. |
| Whorl-related traits | Internode distance | Distance between the neighboring whorls from the youngest to the oldest |
|  | Number of whorls | Total number of the computed whorls |
| Per Whorl Trait | Number of roots | Total number of roots at each whorl across all hierarchy levels |
|  | Level *n* root count | The number of level *n* roots at each whorl |
|  | Total level *n* root length | Sum of the lengths of level *n* roots at each whorl. The length is the skeleton distance from the beginning of the root to its tip. |
|  | Average level *n* root length | Total level *n* root length divided by level *n*  root count at each whorl |
|  | Level *n* root tortuosity | Length of a root (skeleton distance) divided by the Euclidean distance from the beginning to the tip, averaged across all level *n* roots at each whorl. |
|  | Level *n* root thickness | Thickness associated with the skeleton vertices in the root, averaged across all level *n* roots at each whorl. |
|  | Number of level *n* root children | Number of level 2 roots divided by number of level 1 roots for all roots emerged at each whorl. |
|  | Level *n* root tip angle | Angle between the stem direction and the vector from the beginning to the tip of a root, averaged across all level *n* roots emerged at each whorl. |
|  | Level *n* root emergence angle | Angle between the stem direction and the vector from the beginning to 30 vertices along the skeleton of a root, averaged across all level *n* roots emerged at each whorl. |
|  | Level *n* root midpoint angle | Angle between the stem direction and the vector from the beginning of a root to the halfway point of the root, averaged across all level *n* roots emerged at each whorl. |
| Soil Line related traits | Total length above or below the soil line | Sum of the lengths of stem, nodal roots, and lateral roots above or below the soil line |
|  | Root Length Density | the total length of nodal roots and lateral roots per unit soil volume for each centimeter depth under the soil line, considering all roots in a “virtual” soil core - a cylinder whose axis is aligned with the root stem and whose radius covers 95% of all roots in our data set. |
|  | Average length above or below the soil line | Total length above or below the soil line divided by the number of roots above or below the line |
|  | Average stem thickness above or below the soil line | Average thickness of the vertices above or below the soi line along the stem path. |
|  | Stem length | Length of the portion of the stem path above or below the soil line. |
|  | Number of roots | Total number of roots above or below the soil line |
|  | Level *n* root count | The number of level *n* roots above or below the soil line |
|  | Total level *n* root length | Sum of the lengths of level *n* roots above or below the soil line. |
|  | Average level *n* root length | Total level *n* root length divided by level *n*  root count above or below the soil line. |
|  | Level *n* root tortuosity | Length of a root (skeleton distance) divided by the Euclidean distance from the beginning to the tip, averaged across all level *n* roots above or below the soil line. |
|  | Level *n* root thickness | Thickness associated with the skeleton vertices in the root, averaged across all level *n* roots above or below the soil line. |
|  | Number of level *n* root children | Number of level 2 roots divided by number of level 1 roots for all roots above or below the soil line. |
|  | Level *n* root tip angle | Angle between the stem direction and the vector from the beginning to the tip of a root, averaged across all level *n* roots emerged at each whorl. |
|  | Level *n* root emergence angle | Angle between the stem direction and the vector from the beginning to 30 vertices along the skeleton of a root, averaged across all level *n* roots above or below the soil line. |
|  | Level *n* root midpoint angle | Angle between the stem direction and the vector from the beginning of a root to the halfway point of the root, averaged across all level *n* roots above or below the soil line. |
